# Prevalence of ulcerative stomatitis in arbovirus infections in a Brazilian Northeast population

**DOI:** 10.4317/medoral.23865

**Published:** 2020-10-09

**Authors:** Ana Maria Ipólito Barros, Allan Vinícius Martins-de-Barros, Moan Jéfter Fernandes Costa, Pedro Henrique Sette-de-Souza, Eudes Euler de Souza Lucena, Fábio Andrey da Costa Araújo

**Affiliations:** 1 DDS. Resident in Dentistry (emphasis in Oncology). Hospital do Câncer de Pernambuco, University of Pernambuco, Brazil; 2 DDS. Resident in Oral and Maxillofacial Surgery. Hospital Universitário Oswaldo Cruz, University of Pernambuco, Brazil; 3 DDS, MSc. Departament of Dentistry, Federal University of Rio Grande do Norte, Brazil; 4 DDS, MSc, PhD. Associate Professor. School of Dentistry, University of Pernambuco, Campus Arcoverde, Brazil; 5 DDS, MSc, PhD. Associate Professor. Graduate Program in Health and Socioambiental Development, University of Pernambuco, Brazil; 6 DDS, MSc, PhD. Associate Professor. Multicampi Medical Science School, Federal University of Rio Grande do Norte, Brazil

## Abstract

**Background:**

Although there are no population-based studies that support an association, there are reports in the literature of mucocutaneous, vesiculobullous and ulcerated lesions in the oral mucosa in cases of arbovirus infection. The aim of this study is to analyze the prevalence of ulcerative stomatitis in individuals affected by arboviruses in a population of the municipality of Arcoverde, Pernambuco, Brazil.

**Material and Methods:**

1,003 people living in an area assigned to a Primary Health Care Unit were interviewed. A structured questionnaire was used for data collection, with questions about sociodemographic variables, residence conditions, general health information, as well as information about the general signs and symptoms of arboviruses and specifically about oral lesions.

**Results:**

Of the 1,003 individuals interviewed, 815 (81.25%) were infected by one or more arboviruses. Of these, 147 (18%) reported ulcerated oral lesions during arbovirus infections. The association between arbovirus infections and the presence of ulcerated oral lesions was statistically significant (*p* = 0.000).

**Conclusions:**

In these cases, the ulcerated lesions on the oral mucosa appear to be associated with arbovirus infection, especially Chikungunya, although the pathophysiological mechanisms are not defined, and the studies are not sufficient to confirm this association.

** Key words:**Arbovirus infections, Dengue, Chikungunya fever, Zika virus.

## Introduction

Arboviruses are diseases caused by viruses and transmitted to humans mainly by hematophagous arthropods and have become a growing worldwide public health problem. Tropical and subtropical regions possess the social, climatic and environmental conditions favorable to the occurrence of frequent emergence and reemergence phenomena of these viruses (1,2).

In the current Brazilian epidemiological context, the most widely circulated arboviruses are Dengue (DENV), Chikungunya (CHIKV), and Zika (ZIKV) (3). These pathogens are responsible for approximately 95% of human arboviruses reported in Brazil, and their main vectors are hematophagous and anthropophilic mosquitoes of the genera Aedes and Cullex (4).

In 2015, according to the Brazilian Ministry of Health, 1.65 million cases of DENV were reported in the Brazilian territory, with an incidence rate of 813 cases per 100,000 inhabitants. In the same year, 38,499 probable CHIKV cases were recorded. In 2016, 271,824 CHIKV cases and 215,319 probable cases of ZIKV infection were reported, with an incidence of 105.3 cases per 100,000 inhabitants.

Located in the Brazilian Northeast region, the state of Pernambuco has the tenth highest gross domestic product (GDP) in the country and the highest GDP per capita among the Northeastern states. There, between January and December 2016, 65,152 probable DENV cases were reported, one of the highest incidence rates among the Brazilian states. Besides, the record of 48,814 cases of CHIKV and the increasing number of ZIKV cases with laboratory confirmation represent a scenario of simultaneous circulation of the three arbovirus serotypes transmitted by vector mosquitoes, especially Aedes aegypti (5).

These infections have similar clinical characteristics, with signs and symptoms that include fever, myalgia, arthralgia, edema on extremities, headache, retro-orbital pain, conjunctival hyperemia, lymphadenopathy, and maculopapular rash (5,6). Although there are no population-based studies that support an association, there are reports in the literature of mucocutaneous, vesiculobullous and ulcerated lesions in the oral mucosa and oropharynx associated with cases of arbovirus infection (7-9).

During the last outbreak of arboviruses in Arcoverde, PE, patients with ulcerated oral lesions who sought assistance in Family Health Units reported symptomatology compatible with arbovirus infection, according to the Brazilian Ministry of Health’s protocol (6). Thus, the objective of this study was to analyze the prevalence of ulcerated oral lesions, characterized as ulcerative stomatitis, in individuals affected by arboviruses in a population of Arcoverde, PE.

## Material and Methods

It is an epidemiological study of prevalence, being descriptive of the transversal and exploratory type, with a population composed of residents of a region registered by a Family Health Unit (USF) in the city of Arcoverde, PE, which was randomly chosen. The USF is part of the Brazilian health system (SUS), a free health system for all citizens, which provides basic care. Data collection occurred between January and February 2017 and was carried out by six researchers.

Individuals of both genders were included, without age restriction, who had resided in the area assigned to the USF for a period equal to or greater than one year. People who were undergoing radiotherapy and/or chemotherapy treatments, users of orthodontic appliances, users of medications that reduce salivary flow, as well as people with autoimmune disease with repercussions on the oral mucosa, were excluded.

For the sample calculation, the Epi Info 7.04 software was used. The prevalence of 8.8/100,000hab to Chikungunya reported by the Brazilian Ministry of Health was used as a parameter to calculation because it was the most prevalent arbovirus in the period. With a sampling error of 5% and a confidence level of 90%, in a finite population of 3,480 inhabitants, a sample of 850 individuals was obtained. With an increase of 20% to compensate for possible losses in the data collection process, there were 1,020 individuals to be examined (n = 1,020).

**Figure 1:**
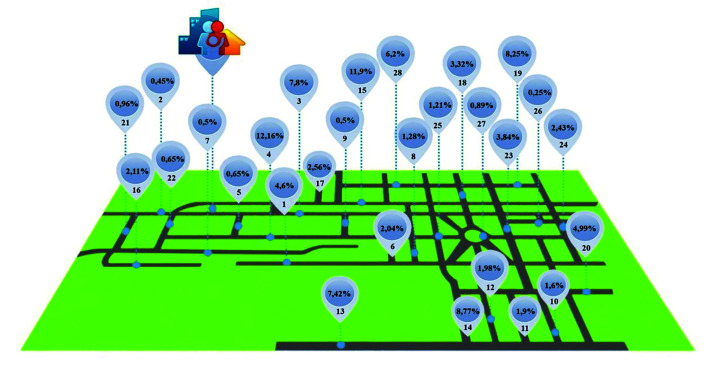
Geographic area assigned to the USF and sample weight assigned per street.

For the selection of houses, a non-probabilistic sampling criterion of stratified quota by geographic area was used. In order to calculate the number of houses per street, a direct search was made of residence records in the e-SUS Primary Care information system, totaling 1,562 houses, distributed among 28 streets. Then, a weight was given to each street according to the number of houses and included individuals in the study. Per street, 2/3 of the houses were visited, following an order of two households visited and one not (Fig. 1).

**Figure 2:**
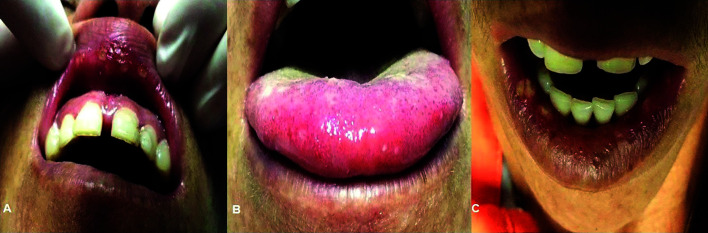
Ulcerative stomatitis in a patient with arbovirus (CHIKV) infection. A: Multiple ulcers in the upper lip; B: Smaller ulcers with erythematous halo in tongue; C: Ulcerated areas covered by fibirnopurulent membrane in lower lip mucosa.

The data collection instrument used was a structured questionnaire with sociodemographic variables, housing questions, general health information, in addition to the general arbovirus signs and symptoms experienced in the past three years and specifically about related oral lesions. Finally, three photos of multiple oral ulcerated lesions on the lip and tongue were shown to the individuals, and they were asked about similarity with the lesions they reported having present (Fig. 2).

The database was built using the SPSS® (Statistical Package for Social Sciences) software version 20.0.0. In order to minimize the typing bias, a double tabulation process was performed by two different researchers, who typed the same questionnaire independently, producing two different databases. Then a comparative evaluation mechanism was performed and, upon verification of the agreement between databases, the statistical analysis began.

Variables were categorized in a dichotomic way to enable a bivariate analysis of the results. The bivariate analysis was performed through prevalence ratios and the chi-square test, with a significance level of 5%. The subjects with missing data for one or more variables were only excluded from the bivariate analysis of the respective missing variables.

## Results

**Figure 3:**
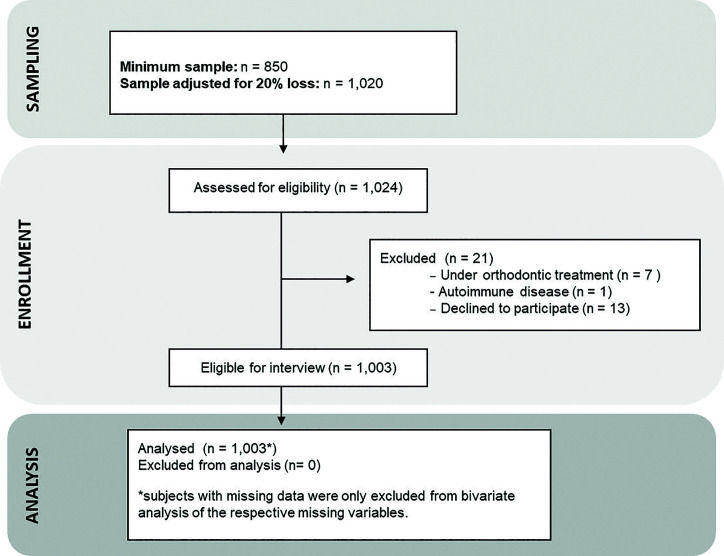
Participant Flowchart of the study.

The participant flowchart is shown in Fig. 3. The study sample was composed of 1,003 individuals aged between 1 and 93 years, of which 64.1% were women. Most participants were “student” (25.0%), followed by "housewife" (12.9%), "retired" (12.5%) and "no occupation" (10.5%). Most of them reported not having habits such as smoking (88.5%) and etilism (81.2%). Detailed individual and household characteristics of the participants are described in Table 1.

Results of the bivariate analysis, as prevalence ratios with confidence intervals and *p-value*, are shown in Table 2.

There was no statistically significant association between sex and arbovirus (*p* < 0.308); however, there was between age and arbovirus (*p* = 0.004).

Regarding household data, 93.6% of individuals had regular garbage collected where they live, and for 59.4%, there was a garbage dump near their home, which was statistically significant (*p* = 0.033). Larvae of the vector mosquitoes were detected in only 6.3% of the houses during the questionnaire. However, there was a statistical significance in the association with this variable with arbovirus (*p* = 0.022).

**Table 1:**
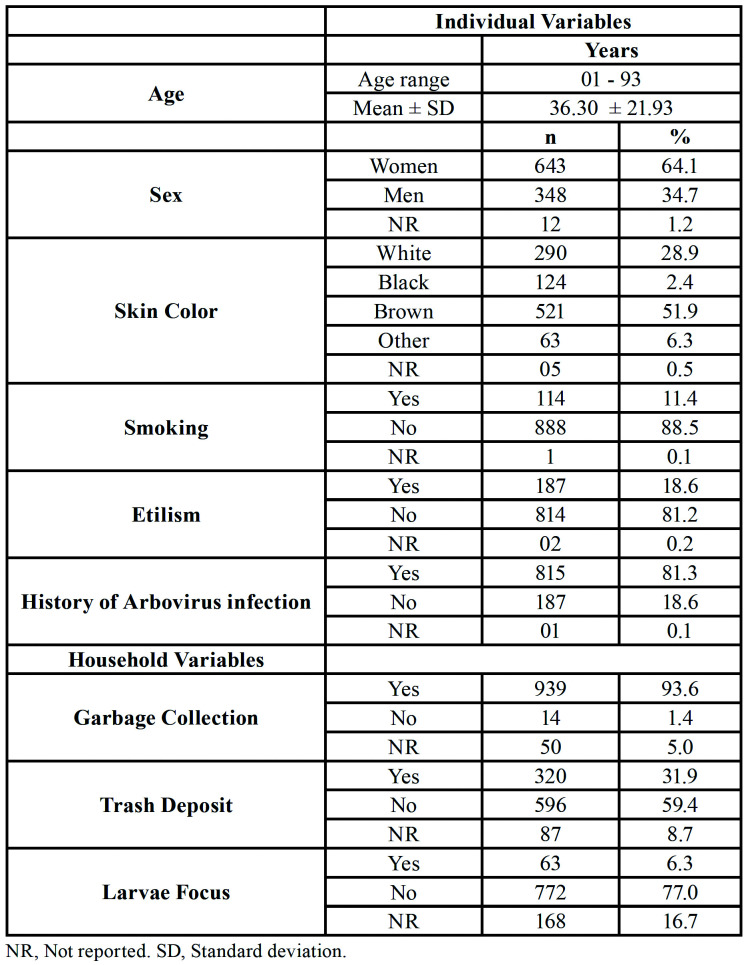
Indivudial and household variables of the subjects enrolled in the study.

**Table 2:**
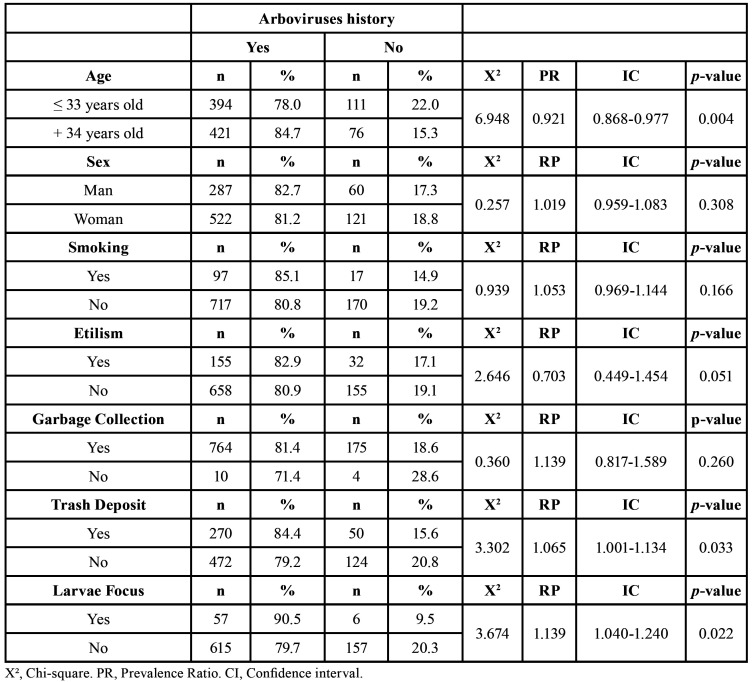
Frequencies, chi-square test, *p-value*, Prevalence Ratio and their respective confidence intervals in the history of Arboviruses associated with independent variables.

Arbovirus infections affected 815 individuals (81.25%). Among them, 53.1% of the individuals reported Chikungunya fever, 15.1% Dengue fever and 4.2% the Zika virus fever, and 9.2% reported having been infected with more than one of these viruses in the past three years. The epidemiologic-clinical diagnosis was the most frequent, with 65.0% of cases studied. Signs and symptoms lasted between 7 and 15 days in 39.8% cases; however, a significant part (23.5%) reported disease persistence symptoms for a period between 30 days and 2 years. Fever, muscle and joint pain were the most frequent signs and symptoms (90.0%), followed by hands and feet swelling, body spots and eye pain reported in more than 50.0% of the cases.

Oral mucosal ulceration was reported in 316 cases, in which 147 reported having oral ulcers during the course of arbovirus infections. In patients who had lesions during infection, 133 (90.4%) reported painful symptoms, and 63% (42.9%) had edema associated. Of these subjects, 124 (84.4%) reported similarity of the lesions from the photos shown in our study. The most frequent ulcer aspects were reddish (n = 48; 33.3%), whitish (n = 38; 26.5%) and yellow (n = 28; 19.0%). Lip lesions appeared in 87 individuals (59.2%), jugal mucosa in 54 cases (36.7%), and 43 cases (29.3%) had a tongue lesion. However, lesions of the gums, palate, mouth floor and throat were also reported in 20 cases (13.7%). Some patients reported oral lesions in more than one site. The association between arboviruses and the presence of ulcerated oral lesions was highly statistically significant (*p* < 0.001), as shown in Table 3.

Some therapeutic protocol was reported in 85 cases (57.8%), in which 35 used some allopathic therapy, such as Triamcinolone Acetonide (Omcilon Orabase®) or Nystatin, and 14 used another protocol, such as bicarbonate, gargle with salt and pomegranate, some having informed of only one or both types of therapy.

**Table 3:**
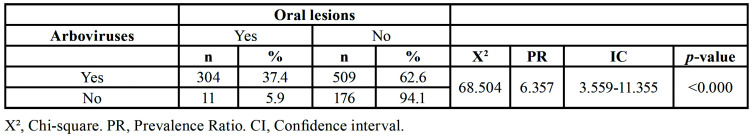
Frequencies, chi-square test, *p-value*, Prevalence Ratio and its respective confidence interval for the presence of oral lesions associated with the independent arbovirus variables.

## Discussion

Arboviruses represent a growing public health problem worldwide. Although many arboviruses are geographically restricted, they have the potential to expand their endemic area due to their capacity for evolution and adaptation (10,11). In addition, climate change, disordered urban growth, poor basic sanitation services such as inadequate garbage collection and consequent garbage accumulation, are risk factors for arboviruses, creating adequate conditions for viruses to proliferate and spread (12,13), which had a statistically significant association in this study.

Chikungunya fever was the most self-reported disease, followed by dengue. These data diverge in a scenario exposed by the Brazilian Ministry of Health, in which in 2014 and 2015, there were more reported cases of dengue than Chikungunya fever. According to the Epidemiological Bulletin of the State Health Surveillance Secretariat, in 2014, there were 589,107 dengue cases reported in the country and 1,688,688 in 2015, of which 110,899 were in the state of Pernambuco.

However, it is possible to observe an evolution in the behavior of Chikungunya fever in Brazil, which has shown a significant increase in suspected cases reported over the years. For example, 3,655 cases were reported in 2014, and 20,901 were reported in 2015, while in 2016, there was an increase to 277,882 cases, of which 151,318 were confirmed by clinical and laboratory tests.

In Brazil, notification about ZIKV infection started in 2016, when it was added to the National Compulsory Notification List of Diseases and Public Health Events. In the same year, 215,319 probable cases of Zika were reported, of which 391 were in the state of Pernambuco. However, according to the Brazilian Ministry of Health, local transmission of ZIKV was confirmed in the country as of April 2015.

The simultaneous circulation of these three viruses is difficult not only for assistance, but also for health surveillance. The similar clinical signs make initial diagnosis difficult and may interfere with notifications. Thus, there may be underreported cases and misinterpreted infections that clinically look like other arboviruses, especially with dengue, which has been overestimated in the notifications (14,15).

Difficulties in performing laboratory tests to confirm a clinical diagnosis is an important bias in arbovirus reported cases. The result found in this study shows that the kind of diagnosis made in most cases (65%) was clinical. Due to the outbreak scenario, the Brazilian Ministry of Health recommends that confirmation of cases should be made by clinical-epidemiological criteria, except in severe cases or those in which the therapeutic protocol must be different, and the specific diagnosis is important (14,15).

However, there is a lot of similarity in the initial symptoms of arboviruses under discussion (10,16). In cases of DENV infection, common symptoms include high fever (> 38ºC), severe headache, retro-orbital pain, myalgia, malaise, nausea, vomiting, asthenia, anorexia, mild arthralgia, and milder hemorrhagic manifestations such as epistaxis, petechiae, and erythema (16,17). Cunha *et al*. (15) and Sales *et al*. (18) describe two phases in clinical CHIKV infection, acute and chronic. The acute phase can be characterized by high fever (> 38ºC), headache, chills, nausea, vomiting, anorexia, myalgia, maculopapular rash, and intense polyarthralgia. The chronic phase is characterized by persistent symptoms such as joint, musculoskeletal and neuropathic pain after recovery time. The most common symptom is joint pain, which may remain for weeks or years after the resolution of the acute condition. ZIKV infections have manifestations such as maculopapular rash, without fever, weak to moderate arthralgia, conjunctivitis, headache, myalgia, and pruritus (15,16).

In this context, signs and symptoms most frequently reported in our study are nonspecific, and common to three arboviruses, such as fever (92.6%), muscle pain (95.1%) and body spots (54.2%). However, most individuals reported arthralgia and edema on extremities, which, although they are not pathognomonic, are more associated with clinical symptoms of CHIKV infection (3).

According to some studies, the clinical manifestation of arbovirus infection has a duration of 7 to 10 days (17,18), similar to the result found in this study. Consequently, it difficult to establish a clinical diagnosis based only on the duration of symptoms. However, in cases in which individuals reported a longer duration of signs and symptoms (30 days to 2 years), CHIKV infection probably occurred in subacute or chronic phase, a clinical pattern that is not associated with other arboviruses (17,19).

Several kinds of mucocutaneous manifestations in arbovirus infections have been documented. The 2017 Health Surveillance Guide from the Brazilian Ministry of Health highlights that other skin manifestations (besides macular or maculopapular rash, common in three arboviruses) are also reported in fever by CHIKV, such as exfoliative dermatitis, vesicular-bullous lesions, hyperpigmentation, and oral ulcers (19). In addition, DENV infections can present gingival bleeding among the weak hemorrhagic manifestations (12).

Different studies (7,20,21) report cases of infected children with CHIKV who presented vesicular-bullous and maculopapular skin manifestations on chest, extremities and face, besides ulcers on the scalp, abdomen, genital and perianal region. Studies showed that oral manifestations are common to CHIKV infection, such as gingivitis, possibly due to the deficiency in oral hygiene associated with arthralgia (22,23), in addition to the burning sensation, hypopigmented macules on the lips, lesions with crusts on the lips and at the mouth's angle, pigmentation on oral mucosa, oral candidiasis, and oral mucosa ulcers (22-27).

According to some studies, the prevalence of ulceration in the oral mucosa ranges between 2% to 12% among individuals with CHIKV fever (25-28). Suryawanshi *et al*. (23), in a descriptive study with 405 suspected cases of CHIKV fever in India, found a prevalence of 9.8% of ulcerated lesions present on the oral mucosa and tongue. Bandyopadhyay and Ghosh (24) report the presence of multiple ulcerations on the tongue, oral mucosa and palate also associated with CHIKV. In the results described here, there is a prevalence of approximately 18% and a highly significant association (*p* < 0.001) between the presence of ulcerated oral lesions and arbovirus infection. Some case reports have described oral manifestations in patients infected with ZIKV, in which ulcerated and bullous lesions were observed in the mucosa and oropharynx (8,9). However, these injuries seem to be more related to CHIKV when we consider that most of the studies found make this relationship.

Similar oral manifestations are caused by other viruses, such as the primary manifestation of infection by Herpes simplex virus type 1 (HSV-1) and infections by Enterovirus, such as echovirus, coxsackievirus A and B and poliovirus. Herpetic gingivostomatitis is the most common pattern of symptomatic primary infection caused by HSV-1 (28).

Since it is a cross-sectional observational study, the results should be analyzed with caution. Although statistical significance is present in the association of some variables, we cannot make a causal inference because a cause-effect limitation inherent to the study design is observed. Besides, another limitation of this study is related to memory bias, once researchers did not make the diagnosis of oral lesions at the moment of data collection. This feature was assessed by a retrospective self-reported questionnaire, and this could lead to some notification mistakes.

In conclusion, ulcerated oral lesions appear to be associated with the manifestations of arbovirus infections, especially CHIKV infection, although they are not sufficient to confirm this relationship. Besides, the pathophysiological mechanisms of lesions related to these arboviruses are not yet well established, characterizing a promising research field.
